# Soft sensor for monitoring biomass subpopulations in mammalian cell culture processes

**DOI:** 10.1007/s10529-017-2408-0

**Published:** 2017-08-07

**Authors:** Paul Kroll, Ines V. Stelzer, Christoph Herwig

**Affiliations:** 1 Research Area Biochemical Engineering, Institute of Chemical Engineering, TU Wien, Gumpendorfer Straße 1a, 1060 Vienna, Austria; 2 Christian Doppler Laboratory for Mechanistic and Physiological Methods for Improved Bioprocesses, TU Wien, Vienna, Austria

**Keywords:** Biomass segregation, Lysed cells, Mammalian cell culture, Monitoring, Soft sensor, Turbidity measurement

## Abstract

**Objectives:**

Biomass subpopulations in mammalian cell culture processes cause impurities and influence productivity, which requires this critical process parameter to be monitored in real-time.

**Results:**

For this reason, a novel soft sensor concept for estimating viable, dead and lysed cell concentration was developed, based on the robust and cheap in situ measurements of permittivity and turbidity in combination with a simple model. It could be shown that the turbidity measurements contain information about all investigated biomass subpopulations. The novelty of the developed soft sensor is the real-time estimation of lysed cell concentration, which is directly correlated to process-related impurities such as DNA and host cell protein in the supernatant. Based on data generated by two fed-batch processes the developed soft sensor is described and discussed.

**Conclusions:**

The presented soft sensor concept provides a tool for viable, dead and lysed cell concentration estimation in real-time with adequate accuracy and enables further applications with respect to process optimization and control.

## Introduction

In accordance with the process analytical technology (PAT) initiative, real-time monitoring of biomass is of great importance as it affects productivity and product quality significantly (FDA 2004). With respect to mammalian cell culture processes, there are numerous technologies and methods available for real-time monitoring of viable biomass which can be used for a multitude of applications such as model-based process control, increasing robustness of processes and higher productivity (Aehle et al. 2011; Biechele et al. 2015; Bogaerts and Wouwer 2003; Frahm et al. 2003; Gnoth et al. 2008; Kroll et al. 2014; Luttmann et al. 2012).

Frequently, biomass is considered to be homogeneous in its composition. In mammalian cell culture processes, however, it was shown previously that segregation of biomass in subpopulations of viable cells, dead cells and lysed cells provides useful information that can be used for process understanding, data evaluation and modeling (Klein et al. 2015; Kroll et al. 2016). With regard to viable and dead cells, several methods are available basing on dielectric and Raman spectroscopy using data driven models (Abu-Absi et al. 2011; Patel and Markx 2008). Lysed cells are of particular interest as they are a source of process-related impurities like DNA and host cell protein in the supernatant. Although lysed cells should be monitored in real-time according to the ICH Q8-R2 guidance (ICH 2009) there are no suitable methods available for control actions and industrial applications. State of the art measurement methods include sampling, an at-line measurement and a calculation step (Klein et al. 2015) which is not applicable for real-time monitoring.

The objective of this study is the development of a simple soft sensor to monitor viable cell concentration (X_VCC_), dead cell concentration (X_DCC_) and the lysed cell concentration (X_LCC_) in a mammalian cell culture process. The presented soft sensor uses simple, robust and common in situ measurements, namely dielectric spectroscopy and turbidity, as inputs and combines them with standard models that are easy to calibrate. Dielectric spectroscopy provides permittivity depending on a measurement frequency and conductivity and turbidity provides an optical density. In order to introduce the soft sensor, which is the combination of a measurement and a model, each measurement signal is analyzed using data from two fed-batch experiments. In addition, the underlying model and its parameters are defined and discussed. Finally, areas of validity and possible applications are presented.

## Methods

### Cultivations

An industrial CHO cell line was cultivated in chemically defined media. In order to investigate biomass segregation in more detail, two fed-batch experiments were performed in a 3.6 l bioreactor system (Labfors 5, Infors, Switzerland). The average working volume was 2 l. The set points for temperature (37 °C), pH (6.8), dissolved oxygen tension (40%) and the partial pressure of carbon dioxide (125 mbar) were chosen in a way to extend the investigated degradation phase. All these process parameters were closed loop controlled. The volume specific aeration rate was 0.015 l l^−1^ min^−1^. The fed-batch experiments were performed using three different feeds, i.e. a glucose feed, a glutamine feed and a feed with limiting components.

### In-process data

Samples were taken every 12 h and analyzed for viable cell concentration, intact dead cell concentration and the concentration of cell debris by an automated image analyzer in triplicates (Cedex HiRes Analyzer, Roche, Mannheim, Germany). Cell debris is defined as particles being smaller than 7 µm. The estimation of the lysed cell concentration was performed according to Klein in triplicates (Klein et al. 2015). All in-process data was used for readjustment of the model parameters.

### Real-time measurements

Soft sensor relevant real-time measurements included (i) the reactor volume V_R_, (ii) oxygen flow into the system F_O2,in_, (iii) permittivity by a dielectric spectroscopy probe (Incyte, Hamilton, Switzerland) and (iv) optical density at 880 nm by a turbidity probe (Dencytee, Hamilton, Switzerland). Settings for the dielectric spectroscopy probe were 1000 kHz. The on-line probes collected data every 6 min. A process information management system (PIMS) (Lucullus 3.2.5, Secure cell, Switzerland) was used for collecting, storing and processing all necessary process data.

### Soft sensor

The real-time process data was used for implementation of a soft sensor, meaning that information about the actual composition of biomass subpopulations could be extracted from multiple real-time measurements for monitoring purposes. The data evaluation and set up of the soft sensor was performed with MatLab (MATLAB R2016a, MathWorks, U.S.A.). All model parameters and shown residuals were estimated using the experimental data of the fed-batches described above.

## Results

### Soft sensor concept

**Fig. 1:**
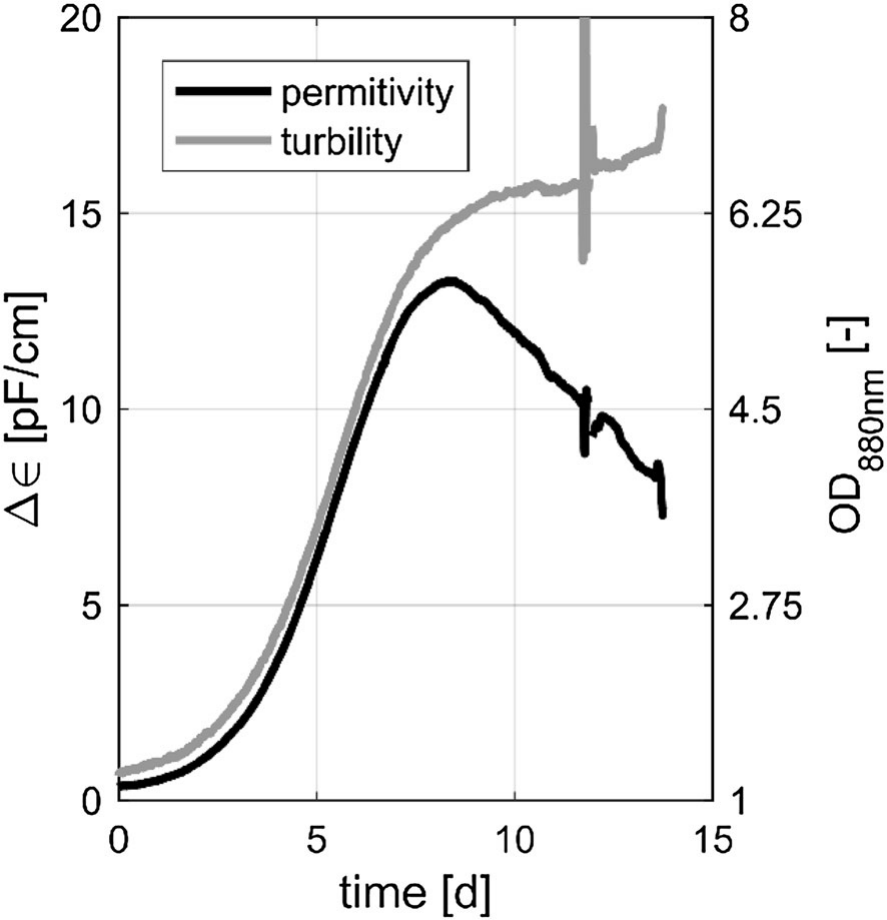
Time course of ∆$\epsilon$ (permittivity) and OD 880 nm (turbidity) over the process time

The soft sensor estimation is based on the observations shown in Fig. 1. The permittivity ($\Delta\epsilon$) and the turbidity (OD_880_) show a similar time course at the beginning. After the 9th day however, a discrepancy between the two signals was observed. The basic hypothesis is that the divergence of these two curves results from a different information content with respect to the previously introduced biomass subpopulations. The turbidity, as a non-specific sum parameter, is known to include information about all absorbing and stray light inducing states such as the biomass subpopulations. In contrast to this, permittivity provides information about the total viable cell volume of intact viable cells.

Target values of the soft sensor are the three biomass states X_VCC_, X_DCC_ and X_LCC_. In a previously published study, it was shown that the investigated biomass subpopulations can be described by three parameters (Kroll et al. 2016), which are the specific growth rate (µ), the specific death rate (k_D_) and the specific lysis rate (k_DL_). In order to determine these three parameters, and consequently the three biomass subpopulations, at least three independent relationships must be established. The first relation bases on the monitoring of X_VCC_ (i.e. permittivity signal), the second relation is based on the turbidity signal used for estimation of X_DCC_ and the third relation is represented by a kinetic model describing X_LCC_ depending on X_VCC_. Details about these three relations will be explained in the following subsections. This combination of methods and technologies is the basis of the soft sensor and allows the real-time estimation of all three target values.

### Real-time estimation of X_VCC_

In principle, any method and technology estimating X_VCC_ can be used for the presented soft sensor concept. Due to this flexibility, the soft sensor concept can be applied for a wide range of set-ups regarding different PAT tools. In this study the X_VCC_ was estimated using a linear correlation between X_VCC_ and firstly the permittivity (see Eq. (1) and secondly the volumetric specific oxygen supply (F_O2_, in V_R_^−1^) (see Eq. (2)), where p_1_, p_2_, p_3_ and p_4_ are parameters that were fitted to the process data by a simple regression analysis (MatLab, ‘regress’). In order to achieve a more robust estimation of X_VCC_, both relationships were taken into account and error-weighted (see Eq. (3)) (Aehle et al. 2010). This is a common approach when there is redundancy.

1 $$ X_{VCC,D} = p_{1} + p_{2} \cdot \Delta \epsilon $$

2 $$ X_{VCC,O2} = p_{3} + p_{4} \cdot \frac{{F_{O2,\,in} }}{{V_{R} }} $$

3 $$ X_{VCC} = \frac{{\frac{{X_{VCC,D} }}{{RMSE_{D} }} + \frac{{X_{VCC,O2} }}{{RMSE_{O2} }}}}{{\frac{1}{{RMSE_{D} }} + \frac{1}{{RMSE_{O2} }}}} $$

The presented relation has an RMSE of 0.61 × 109 cells l^−1^ and is valid as long as (i) the total viable cell volume is linear proportional to the viable cell concentration, (ii) the oxygen-biomass yield and (iii) the k_L,O2_a-value is constant which is satisfied in our case. The estimation of *X*_*VCC*_ could be improved by using additional measurements, which would lead to data redundancy. Furthermore, the re-estimation of the model parameters (p_1_–p_4_) using at-line measurements, such as measurements of X_VCC_ from a cell culture analyzer (Goffaux et al. 2009) could reduce estimation errors. For the aim of this paper the above presented relation (Eq. (3)) is sufficient.

### Information content of the turbidity signal and estimation of X_DCC_

**Fig. 2:**
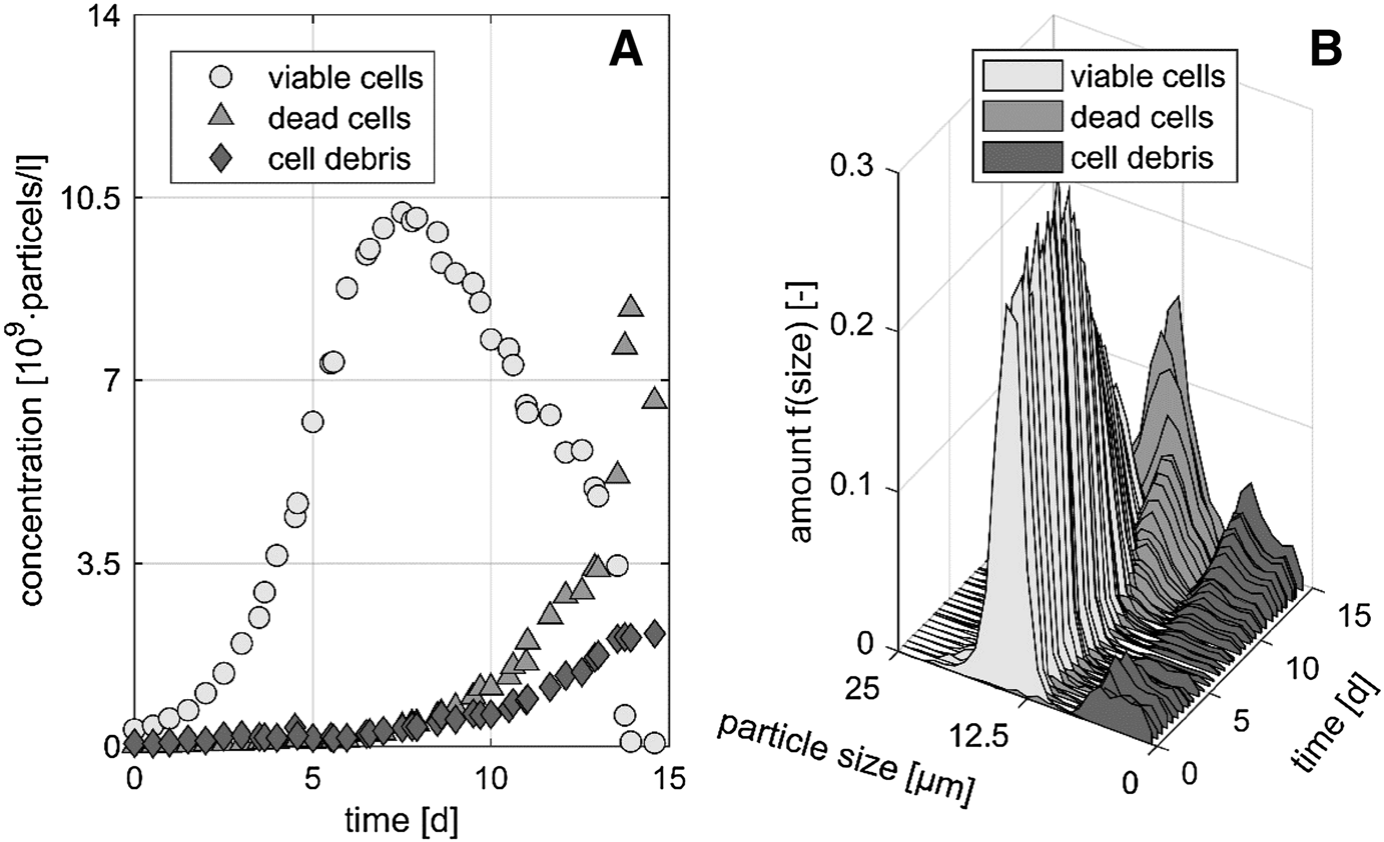
**a** Time course of viable cell concentration (X_VCC_), intact dead cell concentration (X_DCC_) and cell debris concentration over the process time. **b** Proportionate frequency distribution with respect to time and particle size of viable and intact dead cells and cell debris over process time

Figure 2a shows the time course of X_VCC_, X_DCC_ and cell debris concentration for the first fed-batch experiment over process time. It can be seen that the amount of cell debris increases over process time. Cell debris is mainly caused by cell lysis. Figure 2b shows the distribution of viable cells, intact dead cells and cell debris according to particle size and amount of particles with a particular size over process time. It can be seen that the single subpopulations have specific particle sizes and different courses over time. This in combination with different surface characteristics should lead to different extinction coefficients.

The central measurement used by the soft sensor concept is the turbidity. According to the Beer–Lambert law, the total absorbance of a system is the sum of the absorbances of its components. Thus, the absorbance of biomass can be calculated by the absorbance of all considered subpopulations. This leads to Eq. (4), where $\epsilon$_i_ denotes the extinction coefficient, X_i_ denotes the concentration of the ith component and d denotes the path length of the light. Thereby, two different subpopulation sets are considered. In the first case viable cells, intact dead cells and cell debris are taken into account $({\text{i}} \in I = \{ VCC, DCC, cell\, debris\} OD)$. In the second case cell debris is replaced by lysed cells $({\text{i}} \in I = \{ VCC, DCC, LCC\} )$.

4 $$ OD = \mathop \sum \limits_{i \in I} \varepsilon_{i} \cdot d \cdot X_{i} $$

**Fig. 3:**
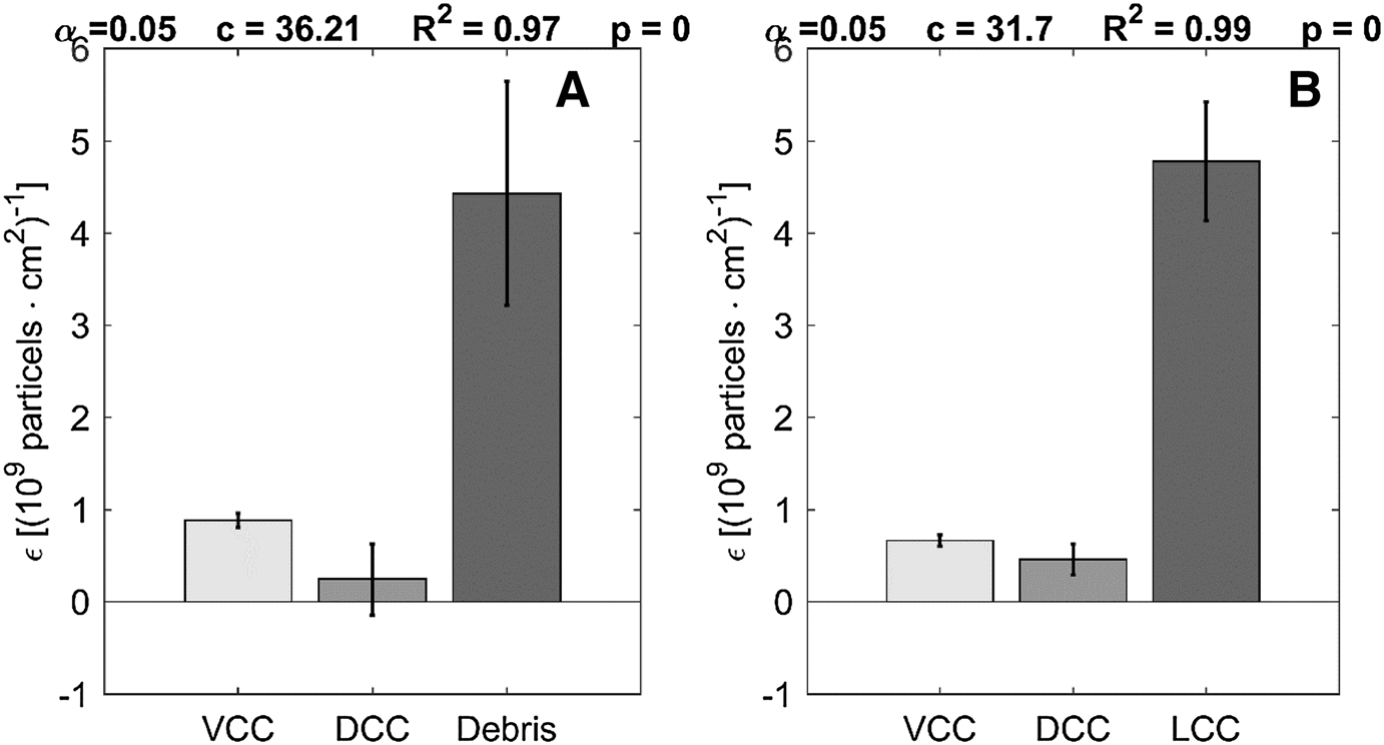
Extinction coefficients for the two investigated models. **a** The first model bases on the segregation of biomass in viable cells, intact dead cells and cell debris ($\epsilon$_VCC_, $\epsilon$_DCC_, $\epsilon$_Debris_). **b** The second model bases on the segregation of biomass in viable cells, intact dead cells and lysed cells ($\epsilon$_VCC_, $\epsilon$_DCC_, $\epsilon$_LCC_)

In order to investigate the turbidity signal in more detail these two absorbance models were compared with respect to the coefficient of determination R^2^, the RMSE, the condition c and the F-value (see Table 1) using experimental data from the first fed-batch experiment. The F-value is in both cases higher than the reference value F_th_ = 2.64 showing that both models are suitable. However, the second model shows better performance with regard to the coefficient of determination, the condition and the RMSE. Furthermore, the extinction coefficients according to the two different models were estimated (see Fig. 3). It can be seen that all extinction coefficients of the second model (Fig. 3b) are significant (confidence level = 0.95). The first model (Fig. 3a) shows a significant extinction coefficient for viable cells and cell debris only. It follows that the second model is more suitable to describe the effects of all three subpopulations. Debris and lysed cells show the highest extinction coefficients. This seems plausible as they are rougher than viable and intact dead cells which leads to more scattered light.

**Table 1:** Statistical parameters for the assessment of the information content of the turbidity signal

	Cell debris based model	Lysis based model
R^2^	0.97	0.99
RMSE [10^9^ cells l^−1^]	0.13	0.04
condition	36.21	31.7
F-value	413	1233

Using the absorbance model basing on lysed cells, *X*_*DCC*_ can be estimated according to Eq. (5),

5 $$\begin{aligned} X_{DCC} \\ = \frac{1}{{ \in_{DCC} }}  \cdot \left[ {OD - OD_{off}\, -\, \in_{VCC} \cdot X_{VCC}\, -\, \in_{LCC} \cdot X_{LCC} } \right] \end{aligned}$$

where OD_off_ denotes the signal offset depending on the media. The presented relationship is valid as long as (i) the extinction coefficients are significant as shown in Fig. 3b, (ii) no other particles such as micro gas bubbles are present in the system and (iii) the Beer–Lambert law is valid. All three conditions were satisfied in the performed experiments.

### Real- time estimation of X_LCC_

In this section the last necessary relation for X_LCC_ is established based on a previously published growth model considering a similar process (Kroll et al. 2016). This ordinary differential equation (ODE) contains the specific lysis rate k_DL_ as parameter that can be assumed to be constant (Kroll et al. 2016). It is a system-describing parameter which depends on lysis influencing parameters such as aeration, power input and reactor geometry. Thus, for a fed-batch process X_LCC_ can be described by Eq. (6) (Kroll et al. 2016). For this purpose, it is necessary to measure feed rates *F*_*in*_ and the reactor volume *V*_*R*_.

6 $$ \frac{{dx_{LCC} }}{dt} = k_{DL} \cdot x_{VCC} - \frac{{F_{in} }}{{V_{R} }} \cdot x_{LCC} $$

### Performance of the soft sensor

**Fig. 4:**
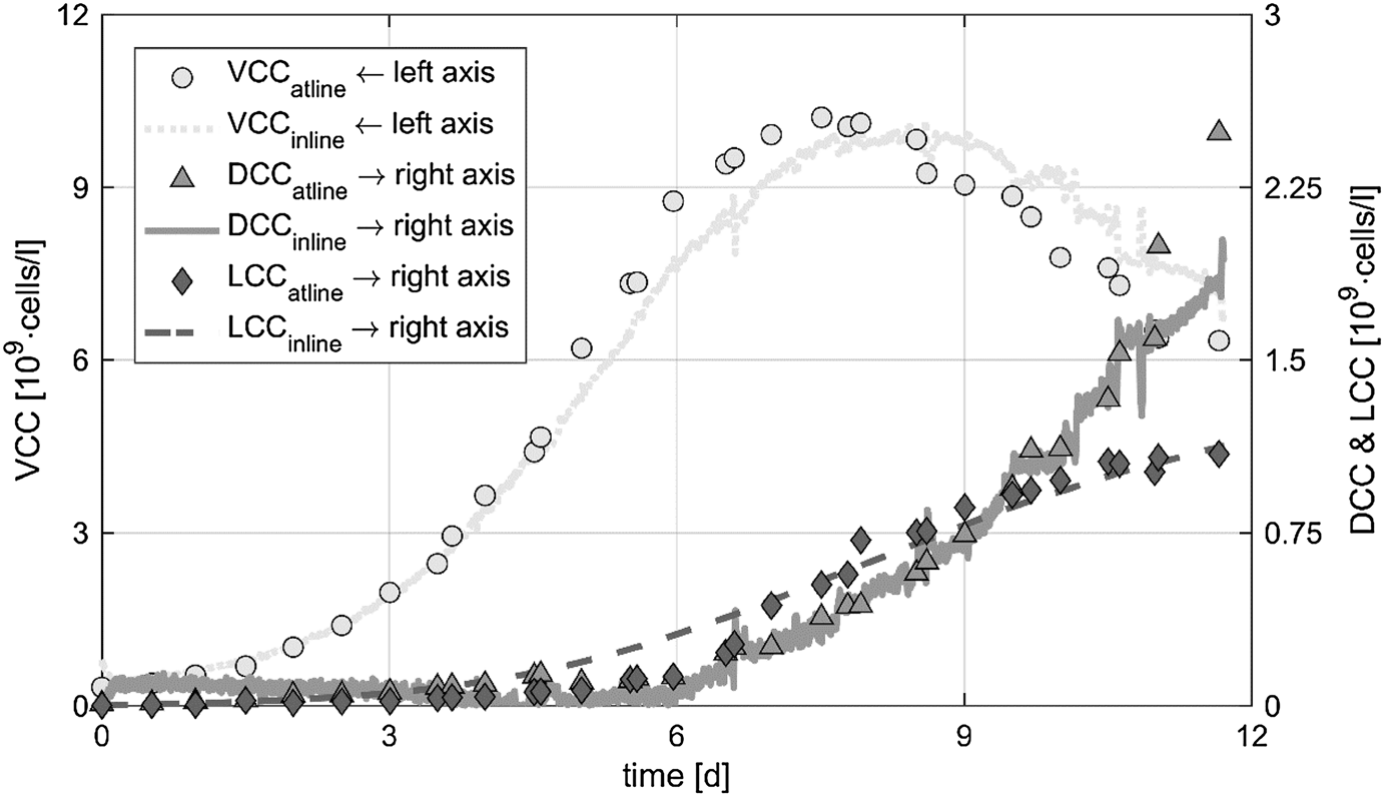
At-line measured and real-time estimated X_VCC_, X_DCC_ and X_LCC_ over a fed-batch process

Combining the three relationships described above, all biomass states (X_VCC_, X_DCC_ and X_LCC_) can be estimated using continuous measured signals. This soft sensor bases on three equations and nine parameters. The order of implementation is: (i) real-time estimation of X_VCC_ according to Eq. (3) which is independent of the other biomass subpopulations, (ii) estimation of X_LCC_ basing on the previously determined X_VCC_ using Eq. (6) and (iii) the calculation of X_DCC_ using the measured turbidity based on Eq. (5) and the calculated values for X_VCC_ and X_LCC_. All parameters can be calibrated separately by historical or in process data. The single equations are simple and easy to implement. Thus, the presented soft sensor was set up in accordance to the basic principle of process engineering being as simple as possible and as accurate as necessary. Figure 4 shows the at-line measurements and the soft sensor estimations of all investigated biomass subpopulations. With respect to accuracy of reference methods, all states could be determined with an adequate RMSE which is comparable to deviations of the reference measurements (see Table 2).

**Table 2:** RMSE and NRMSE of X_VCC_, X_DCC_ and X_LCC_ for two independent experiments (1 and 2)

Fermentation	X_VCC_		X_DCC_		X_LCC_	
	1	2	1	2	1	2
RMSE [10^9^ cells l^−1^]	0.61	0.46	0.12	0.14	0.08	0.17
NRMSE [−]	0.1	0.07	0.24	0.27	0.18	0.37

## Discussion

The presented soft sensor is based on several assumptions: (i) the oxygen biomass yield is constant, (ii) the k_L,O2_a is constant, (iii) the cell specific volume is constant, (iv) the extinction coefficients of the biomass subpopulations are significant and constant, (v) no other absorption active particles such as micro gas bubbles are present, (vi) the turbidity is not saturated and (vii) the specific lysis rate is constant. These assumptions can be reduced by the use of additional methods. For example, a method for the real-time estimation of model parameters could expand the validity area of the soft sensor (Bogaerts and Wouwer 2003; Goffaux et al. 2009). The observed design space for the soft sensor was: X_VCC_: 0.03–10.5 [10^9^ cells l^−1^], X_DCC_: 0–2.4 [10^9^ cells l^−1^], X_LCC_: 0–1.2 [10^9^ cells l^−1^]. Furthermore, the concept of the soft sensor is easily transferable to different measurement methods and helps to improve process understanding and control strategies.

The developed soft sensor represents a method for estimating viable, dead and lysed cells of biomass in mammalian cell culture processes in real time. The real-time estimation of lysed cells in mammalian cell culture processes has not been reported so far to the knowledge of the authors. The soft sensor concept bases on a careful selection of model equations, parameters and simple in situ measurements. The time-resolved information obtained by the soft sensor with respect to the subpopulations can be used for (i) reactor quantification with respect to cell damage and for (ii) each biomass related control action such as real-time identification of termination criteria depending on viability or lysed cell concentration. This could have a direct impact on product quality and on productivity. Especially the influence of lysis on the downstream would be an interesting point for further studies.

## Mathematical notations

*d*: Path length of light
*F*_*O*2, *in*_: Oxygen flow in the reactor
*k*_*DL*_: Specific lysis rate
*OD*: Optical density
*p*_*j*_: Regression coefficients
*RMSE*_*i*_: Root mean square errors
*V*_*R*_: Reactor working volume
*X*_*i*_: Biomass state i
$$\Delta\epsilon$$: Delta permittivity
*ɛ*_*i*_: Attenuation coefficient of the species *i*
